# The effects of an acute exercise bout on GH and IGF-1 in prediabetic and healthy African Americans: A pilot study investigating gene expression

**DOI:** 10.1371/journal.pone.0191331

**Published:** 2018-01-19

**Authors:** Nathaniel T. Berry, Monica Hubal, Laurie Wideman

**Affiliations:** 1 University of North Carolina at Greensboro, Greensboro, NC, United States of America; 2 George Washington University Milken Institute School of Public Health, Washington, D.C., United States of America; 3 Children's National Medical Center, NW, Washington, D.C., United States of America; Vanderbilt University, UNITED STATES

## Abstract

The incidence of pre-diabetes (PD) and Type-2 Diabetes Mellitus (T2D) is a worldwide epidemic. African American (AA) individuals are disproportionately more likely to become diabetic than other ethnic groups. Over the long-term, metabolic complications related to diabetes result in significant alterations in growth hormone (GH) and insulin-like growth factor-1 (IGF-1). Considering the limited exercise-related studies in the area of gene expression changes with disease progression, the objective of this study was to examine differences in exercise-induced gene expression related to the GH and IGF-1 pathways in peripheral blood mononuclear cells (PBMCs) of healthy (CON) and PD AA individuals. Design: Ten subjects [5 PD (age = 35±9.3 yr, BMI = 32.1±4.0, FBG = 101.8±1.3 mg/dl) and 5 CON (age = 31±9.4 yr, BMI = 29.4±5.2, FBG = 82.8±9.7 mg/dl)] had blood drawn for RNA isolation prior to exercise (Pre), immediately following acute moderate intensity exercise on a treadmill (Post-1), 6-hours post (Post-6), and 24-hours post (Post-24). Isolation of mRNA from PBMCs was performed using ficoll separation, while the profiling of mRNA expression was performed using Illumina beadchip arrays with standard protocols. Scan results were statistically analyzed for a specific list of genes related to GH and IGF-1. GH and IGF-1 protein levels were also assessed in each sample. To address issues of normality, all GH and IGF-1 data were log-transformed prior to analysis. Statistical significance was set at p<0.05. Results: Group differences for GH2 variant 2 (p = 0.070) and GH2 variant 3 (p = 0.059) were coupled with significant alterations in IGF-1 mRNA over time (p = 0.024). A significant interaction between group and time was observed for GHRH mRNA (p = 0.008). No group differences were observed in GH AUC (p = 0.649), ΔGH (p = 0.331), GH_rec_ (p = 0.294), or IGF-1 AUC (p = 0.865), representing a similar exercise-induced GH and IGF-1 response for both groups. Conclusions: Analysis of GH and IGF-1 related-gene expression indicates that mild elevations in fasting blood glucose and exercise-induced alterations in gene expression are impacted by the prediabetic state.

## Introduction

The number of individuals diagnosed with type-2 diabetes mellitus (T2D) has continued to increase over the past several decades [1]. It was estimated that 422 million adults were living with diabetes in 2014 and that the age-standardized global prevalence of diabetes has nearly doubled since then [2]. The complications associated with T2D include higher risk of heart attack, stroke, kidney disease, and increased rates of hospitalization, as well as premature death [2]. Given the dire health consequences of long-term mismanagement of T2D and the significant medical costs associated with T2D complications, significant resources have been focused on disease management.

There is significant inter-individual variation in disease progression that occurs over time and impacts different systems across levels of physiological organization. It should be remembered that lower levels of physiological organization (i.e. gene expression) work on faster timescales and are more flexible than markers at higher levels of organization (i.e. proteins). This physiological organization contextualizes how changes and adaptations at the gene expression level may be more similar between PD and T2D, whereas time-corresponding protein levels may be more similar between PD and CON. Since disease progression occurs on a continuum, understanding how physiological responses at various levels of biological organization differ throughout the disease continuum may provide important information for future methods of detection and treatment.

It is well known that the degree of insulin resistance necessary to cause persistent hyperglycemia may be present for extended periods of time prior to a clinical diagnosis of T2D. Depending when measurement occurs throughout the continuum of disease progression, these adaptations may be coupled with a variety of additional physiological changes including system wide alterations in several other endocrine pathways. Individuals in the prediabetic (PD) state are identified as having a glycosylated hemoglobin (HbA_1C_) between 5.7–6.4%, a fasting blood glucose (FBG) between 100–125 mg/dl, or an oral glucose tolerance test (OGTT) between 140–199 mg/dl [2]. These individuals are encouraged by physicians to manage their blood glucose with lifestyle modifications as the progression of T2D is exacerbated by obesity and an inactive lifestyle [3]. Pre-diabetic individuals are typically advised to decrease their fat and energy intake and lose 5–10% of their body weight through exercise and dietary modification [2]. Approximately 70% of individuals with pre-diabetes develop T2D [4] and an estimated 35% of Americans over the age of 20 are pre-diabetic, while approximately half of the individuals over the age of 65 are affected by diabetes [5]. While testing fasting blood glucose and measuring HbA_1c_ may provide an indication of disease onset, the progressive nature of the pathophysiology inherent in the development of T2D suggests that many metabolic alterations precede overt disease.

During rest, differences in the activation of signaling pathways exist between individuals with T2D and healthy controls [3, 6]. While the signaling alterations in full blown diabetes have been extensively described, the timing of these changes with relation to disease progression are not clearly elucidated and even less is understood regarding the relationship between disease progression (as in PD) and the effect of exercise on altering various signaling pathways. Exercise has well established effects on insulin-related signaling pathways in T2D [2, 3] and exercise training results in a re-sensitization of the endocrine system in these individuals that beneficially effects hormonal regulation of many different physiological functions [2, 3]. Comparisons between the effects of acute and chronic aerobic and strength training on blood glucose management and insulin sensitivity in T2D are provided elsewhere [7], but can generally be summarized as beneficial. While specific questions regarding whether or not the exercise-induced responses of PD individuals are more like healthy individuals or more like T2D individuals may be posed, these relationships should be considered to be on a continuum between healthy and T2D individuals; remembering that lower levels of physiological organization often work on faster timescales and are more flexible than markers at higher levels of organization. While the measurement of messenger ribonucleic acid (mRNA) responses to exercise have predominantly been performed using muscle tissue samples, it has been suggested that peripheral blood mononuclear cells (PBMC) may offer an effective whole-body measure of response [8]. Both aerobic [9–11] and resistance exercise [12] significantly alter PBMC gene expression. While there is a small literature base in the area of PBMC and exercise-induced alterations in gene expression, there are very few articles investigating changes in these responses in diseased individuals. However, of the limited studies available, PBMC gene expression has been shown to reflect the pathophysiology of diabetes [8], but this study did not include exercise. While several DNA single nucleotide polymorphisms (SNPs) have been linked to the development of T2D in genome wide association studies [13–16], very little research has focused on discerning the deviations in various signaling pathways and the downstream metabolic alterations that occur early in disease. Understanding how these pathways respond to physiological stimuli such as exercise, which is known to benefit individuals with T2D, during the early stages of disease may help to identify key molecules in the pathway that could be targeted for further investigation. While some of the T2D related alterations in thyroid, adrenal, and gonadal function are reversed by insulin replacement therapy, glycemic control does not normalize endocrine function [17].

The link between growth hormone (GH) and T2D has been well established and either over-synthesis of GH or exogenous GH supplementation can result in the development of T2D [18]. GH secretory changes are observed across ages, between genders, and in various disease states [17], including diabetes [19]. In addition, growth hormone and IGF-1 play significant roles in body composition and have been associated with alterations in lean body mass and fat mass [20] which further complicates T2D [19].

Asplin et al. [19] previously reported altered pulse frequency and interpulse GH concentration with hypothalamic dysfunction in diabetics. These alterations result in deviations from the normal, “healthy,” interaction dominant dynamics across levels of physiological organization. Knowing at what point during disease progression these alterations begin to occur could provide vital information for clinicians and researchers. For instance, these alterations may be the result of upregulated promotional pathways and associated transcripts, down regulation of inhibitory pathways, or both. Either way, better understanding these dynamic relationships could prove critical to our ability to assess, diagnosis, monitor, and treat disease. Both lean body mass and fat mass are significantly impacted by GH [20] and a 7–8% reduction in lean body mass (LBM) has been observed in GH deficient individuals compared to healthy controls [21–23]. Meanwhile, fat mass accumulation has also been associated with GH deficiency [21, 24, 25]. In addition to decreased LBM and increased fat mass, analysis of fat mass distribution has shown that fat mass accumulates preferentially in the abdominal area. These individuals also have increased risk of cardiovascular events, T2D, mortality, and morbidity [26, 27]. Obese individuals also have overall reductions in GH output [28] and all of this taken together suggests an intricate relationship between body composition, GH, and alterations in metabolism that promote a negative shift in the overall health of an individual.

Given the current understanding of the relationships between T2D, GH, IGF-1, coupled with the lack of exercise-related studies in the area of gene expression, this study aimed to investigate the effects of an acute bout of moderate-intensity exercise on PBMC gene expression between healthy and PD individuals. Furthermore, because certain groups such as African Americans (AA) are more likely to become diabetic than others [5], this study focuses specifically on comparing the effects of an acute bout of moderate-intensity exercise on PBMC gene expression between healthy and PD AA individuals. Defects in insulin secretion, insulin action, or both, result in hyperglycemia, which when sustained over extended periods of time, results in damage, dysfunction and eventually failure of various organs. We expected that the exercise-related profiles of the GH and IGF-1 proteins would be similar between the two groups but that the expression of GH and IGF-1 related mRNA would differ between the two groups in response to the exercise stimulus. To further our understanding of early changes in pre-diabetic individuals that may be influenced by exercise, we also investigated potential gene expression differences for related metabolic and inflammatory pathways.

## Material and methods

### General overview

Individuals were screened over the phone and those who met the initial screening criteria were scheduled to come into the laboratory for an additional screening process. Upon arrival to the laboratory screening visit, informed written consent was obtained and anthropometric measures were taken prior to a fasted blood draw. Following the screening visit, individuals reported twice more; once for baseline testing and a second time to complete an acute submaximal exercise bout. During the exercise visit, blood samples were taken prior to beginning exercise (Pre), immediately post (Post-1), 6-hours post (Post-6), and 24-hours post (Post-24). This study was approved by the Institutional Review Board at the University of North Carolina at Greensboro (09–0174, PI: Wideman). All data pertaining to this manuscript are available at Gene Expression Omnibus (GEO) under acquisition number GSE101931.

### Initial phone screening

Individuals were recruited from a larger study. Participants who expressed interest in this portion of the study were initially contacted by phone and after the study was explained, if they were still interested and met screening inclusion criteria, a laboratory visit was scheduled. A member of the research team determined if the individual met all of the screening inclusion criteria; age 18–45 years, self-identified as African American, no known blood glucose issues and self-reported BMI>19 and <36 kg/m^2^. Fasting blood glucose was confirmed at the laboratory screening visit. Any individual with a FBG >126 mg/dl, with known active disease (cardiovascular, metabolic, or pulmonary), BMI>36 kg/m^2^ or with orthopedic limitations were excluded from the study. Other exclusions included: acute or chronic health conditions, medications for cardiovascular disease, mental health, endocrine, infectious conditions, or a history of cancer treatment. The first ten individuals who expressed interest in this study were scheduled for a laboratory visit.

### Laboratory screening visit

Upon arriving in the laboratory, all individuals provided informed written consent. Each individual was then asked to complete a detailed family history and medical questionnaire (S1 File) and a physical activity questionnaire [29]. Height and weight were assessed and BMI calculated to confirm that each individual met the inclusion criteria. A blood draw (5ml) was taken following a 10-hour fast to assess FBG, insulin, and HbA_1C_. The first 5 individuals with FBG between 100–125 mg/dl and without active disease or orthopedic limitations were enrolled into the PD group. Similarly, the first 5 individuals with FBG of less <100 mg/dl and without disease or orthopedic limitations were enrolled into the healthy (CON) group. Of the first ten individuals who expressed interest in this study, all ten met the inclusion criteria; five of which met the PD criteria while five were enrolled into the study as healthy controls. Of these ten individuals, none dropped out and none were removed from the study for any reason.

### Baseline assessment visit

Participants were asked to return to the laboratory to have a comprehensive body composition assessment completed and were asked to perform a submaximal exercise stress test. Body composition was performed via Dual-energy X-ray absorptiometry (DXA) (GE Lunar Prodigy). The submaximal exercise protocol utilized 3 minute stages on a treadmill (Quinton Q-Stress TM65). The test was terminated when the participant reached 85% of his/her heart rate (HR) reserve (HRR); oxygen uptake (VO_2_) was assessed throughout and maximal VO_2_ (VO_2max_) was extrapolated.

### Acute submaximal exercise visit

Participants returned to the lab at least 48 hours after the baseline assessment visit to perform an acute submaximal exercise bout. All participants were asked to eat a standardized breakfast of juice and toast, approximately 1.5–2 hours prior to arriving at the lab. Upon arrival in the lab, subjects completed a 3-day dietary recall, and resting blood pressure (BP), HR, and resting pre-exercise blood sample were obtained. Subjects completed a 60-minute exercise bout at moderate intensity (60% VO_2_ reserve) on the treadmill. HR, BP, and a rating of perceived exertion (RPE) was taken every 15-minutes. Methodologically, this 60-minute moderate intensity exercise bout was chosen in order to meet the American College of Sports Medicine (ACSM) guidelines for weight management. Workload was adjusted accordingly and to assure that each individual could complete the 60-minute exercise bout. Immediately following exercise, an additional blood sample was taken (Post-1). Subjects returned to the lab 6 hours later (Post-6) (at least 2-hours post-prandial) to complete another blood draw. The following morning, individuals ate the same standardized breakfast as they had the previous day (average total calories: 207±119kcal; protein: 10.8±3.0%; carbohydrate: 81.5±6.6%; fat: 8.2±2.4%;) and returned to the laboratory for a fourth blood sample (Post-24) taken 24 hours after the cessation of the exercise bout.

### Blood processing & RNA preparation

A portion of each blood sample collected during the acute submaximal exercise visit was utilized to analyze the response of plasma proteins. The remainder of the blood was processed and PBMCs were isolated using ficoll separation. Total RNA was isolated from PBMCs using a Qiagen kit that was specifically designed for blood extractions and stored at -80°C until analysis.

### Microarray procedures & analysis

Quality controls included adequate amplifications, thresholds for appropriate scaling factors, detection p values and internal controls for RNA integrity. Any sample not meeting all QC values after processing through microarray was reprocessed from the original total RNA samples, after the integrity of the RNA samples was verified by agarose gel electrophoresis and imaging. Two round amplification of total RNA was done using the Illumina HumanHT-12 v4.0 Expression BeadChip Array for gene expression analysis (Illumina Inc., San Diego, CA). Thirty μg of biotinylated complementary RNA (cRNA) from the second amplification round was hybridized to Illumina microarray techniques. Data processing of resultant iDAT files is described below. Average normalization was done within the BeadStudio module of Illumina’s GenomeStudio. Expression levels were imported directly into Partek Genomics Suite (Partek Inc., St. Louis, MO) for statistical analysis.

A small sample of RNA was quantified by reverse transcription polymerase chain reaction (RT-PCR) to ensure that the RNA was intact and free from DNA contamination. A second sample was quantified by RT-PCR after microarray analysis as a cross-validation for any significant changes detected by the microarray analysis.

### RT-PCR procedures

Once microarray analysis was completed, the genes with the greatest fold change from baseline to post-exercise as well as several additional genes of interest were investigated. Once identified, RT-PCR was completed to verify the findings from the microarray analysis. Quality control for RT-PCR was performed using GAPDH, while analysis of PKC2/PRKCZ, GCGR2, RXRG, HSPA1B, SREBP1, IL8, and MAPK7 were performed using the stored RNA sample. For the RT-PCR, primers that lie on either side of an exon/intron boundary were chosen for those genes that were selected for further validation. Assay kits from Applied Biosystems (ABI) were used: GAPDH, Hs02758991_g1; PRKCZ, Hs00177051_m1; GCGR, Hs01026189_g1; RXRG, Hs00199455_m1; HSPA1B, Hs01040501_sH; SREBF1, Hs01088691_m1; IL8, Hs00174103_m1; JUN, Hs01103582_s1; MAPK7, Hs00611114_g1. RT-PCR was performed with 1μg of RNA from the stored samples. RNAse-free DNAse I was used to digest any DNA that was co-isolated with the RNA. Having used primers that cross introns further served to ensure that a false positive signal is not obtained. An RNAse-treated control samples was also subjected to RT-PCR to ensure that amplification arose from RNA. A commercially available kit was used for RT-PCR and a control RT-PCR reaction was run with each sample using the ubiquitously expressed rp49 gene in order to ensure that the quantity of RNA and the efficiency of the RT-PCR amplification was the same in each sample.

### Bioassays

All bioassays were performed on serum samples. Growth hormone and IGF-1 were assessed using enzyme linked immunosorbent assays (ELISA) from RayBiotech, Inc. and IDS (Immunodiagnostic systems), respectively. Kits from R&D Systems (Minneapolis, MN) were used to assess TNF-α and IL-6 respectively while fasting blood glucose was assessed using a colorimetric assay kit (Cayman Chemicals, Ann Arbor, Michigan) and insulin was assessed using an ELISA (Mercodia, Winston-Salem, NC).

### Data analysis and statistics

Resultant gene lists were analyzed to determine differences in gene expression between groups at baseline and following the acute exercise stimulus, using repeated measures analysis of covariance (ANCOVA). Due to the known effects of increased body composition to alter GH secretory patterns as well as the relationships between body composition and T2D, body composition (%fat) was used in the analysis to control for these differences. Statistical significance was evaluated using multivariate permutation tests that controlled for the false discovery rate [30]. Lists of probe sets with differential expression between groups over time were analyzed using Ingenuity Pathway Analysis (IPA) software, which generates novel networks of molecule interactions and maps expression data onto well-established canonical pathways.

All statistical procedures related to bioassays and subject demographics were performed in R statistical software [31]. Log-transformations were performed on all GH, IGF-1 IL-6, and TNF-α protein data to correct for violations of normality. Area under the curve (AUC) was calculated using the Pre, Post-1, Post-6, and Post-24 time points. The ΔGH, ΔIL-6, and ΔTNF-α values were calculated as the difference from Pre to Post-1 while recovery (i.e. GH_rec_, TNF-α_rec_, and IL-6_rec,_) was calculated as the difference between Post-1 and Post-6. An ANCOVA was used to assess mean differences between groups after controlling for body composition (%fat) while two-sample t-tests were used to determine group differences between measures taken during the baseline assessment testing session. Significance was determined *a priori* (p<0.05) for all statistical tests.

## Results

Subject characteristics are shown in Table 1 and this table also provides values obtained during the baseline testing session. As expected based on screening criteria, comparison of FBG values from the screening visit indicates a significant difference between pre-diabetic (PD) and control (CON) (t = 4.327, *df* = 8, p = 0.002) while differences in the mean values of HbA_1C_ indicate a near significant group effect (t = 2.298, *df* = 8, p = 0.051). In both PD and CON, only 1 individual per group reported having a first-degree relative with T2D while 2 and 4 individuals in the PD and CON groups, respectively, reported having second-degree relatives with T2D. Response to acute moderate intensity exercise is summarized in Table 2 for the PD and CON groups. No statistically significant differences were observed for any of the variables reported in Table 2. Genes of interest, and all pertinent p-values for interactions and main effects of group and time are provided in Table 3. Post-hoc analyses indicating the fold differences for significant interactions and main effects are provided in Table 4.

**Table 1:** Subject demographics organized by group.

	CON		PD	
	Mean	(±SD)	Mean	(±SD)
**Age**	31	(±9)	35	(±9)
**Wt (kg)**	84.4	(±26.0)	100.1	(±11.2)
**Ht (cm)**	167.7	(±12.1)	176.9	(±5.8)
**BMI (kg/m**^**2**^**)**	29.4	(±5.2)	32.1	(±4.0)
**FBG (mg/dl)**	82.8	(±9.7)	101.8	(±1.3)*
**HbA**_**1C**_ **(%)**	5.7	(±0.3)	6.0	(±0.1)†
**Insulin (ng/ml)**	6.9	(±1.7)	7.6	(±4.2)
**Sag. Diam. (cm)**	24.1	(±5.3)	27.1	(±4.1)
**Waist (cm)**	86.6	(±16.1)	98.6	(±6.1)
**Hip (cm)**	101.6	(±23.8)	113.8	(±8.1)
**Thigh (cm)**	61.6	(±4.3)	61.8	(±8.2)
**Fat (%)**	34.5	(±6.9)	32.4	(±12.4)
**Lean Mass (kg)**	43.9	(±14.5)	65.2	(±12.0)
**Fat Mass (kg)**	37.9	(±26.1)	31.6	(±13.7)

Presented as mean (±SD).

*Denotes significant group difference (p<0.05)

†Denotes group difference (p<0.10).

BMI–body mass index; FBG–fasting blood glucose; HbA_1C_ - glycosylated hemoglobin; Sag. Diam.–sagittal diameter; Waist–waist circumference; Hip–hip circumference; Thigh–thigh circumference.

**Table 2:** Individual exercise responses separated by group.

		CON		PD	
		Mean	(±SD)	Mean	(±SD)
**BP (mmHg)**	**Rest**	127/82	(±13/10)	123/71	(±16/14)
	**Max**	137/85	(±19/10)	144/83	(±26/15)
**HR (bpm)**	**Rest**	72	(±10)	70	(±11)
	**0–20**	146	(±15)	144	(±10)
	**20–40**	154	(±13)	148	(±12)
	**40–60**	156	(±13)	147	(±11)
**RPE**	**5–10**	11.3	(±2.2)	10.8	(±2.5)
	**15–30**	11.8	(±2.6)	12.1	(±2.6)
	**45–60**	12.0	(±2.3)	13.0	(±2.4)
	**O_2_**	110	(±44)	136	(±26)
	**Kcal**	549	(±219)	680	(±128)

Presented as mean (±SD). O_2_ –total oxygen consumption over the course of the hour long exercise bout–measured by Parvo Medics TrueOne 2400; Kcal–estimated kilocalorie expenditure calculated based on oxygen consumption; Heart rate–measures taken every five minutes and reported values are averages of those measures per denoted time frame; RPE–reported values are averages of measures taken denoted time points.

**Table 3:** Gene symbols and p-values for interactions, group, and time effects.

Symbol	Definition	Int.	Group	Time
GH1	Growth hormone 1, transcript variant 3		0.263	0.843
GH1	Growth hormone 1, transcript variant 5		0.117	0.799
GH2	Growth hormone 2, transcript variant 2		0.926	0.582
GH2	Growth hormone 2, transcript variant 3		0.059	0.722
GH2	Growth hormone 2, transcript variant 2		0.204	0.354
GH2	Growth hormone 2, transcript variant 4		0.071	0.335
GHRH	Growth hormone releasing hormone	0.008		
GHRHR	Growth hormone releasing hormone receptor, transcript variant 2		0.787	0.568
GHRHR	Growth hormone releasing hormone receptor, transcript variant 1		0.869	0.446
GHRHR	Growth hormone releasing hormone receptor, transcript variant 2,		0.678	0.873
SST	Somatostatin		0.657	0.095
SSTR1	Somatostatin receptor 1		0.504	0.342
SSTR2	Somatostatin receptor 2		0.020	0.986
SSTR3	Somatostatin receptor 3		0.672	0.270
SSTR4	Somatostatin receptor 4		0.359	0.910
SSTR5	Somatostatin receptor 5		0.530	0.694
IGF1	Insulin-like growth factor 1 (somatomedin C)		0.500	0.024
IGF1R	Insulin-like growth factor 1 receptor		0.544	0.352
IGFBP1	Insulin-like growth factor binding protein 1		0.850	0.985
IGFBP2	Insulin-like growth factor binding protein 2		0.794	0.226
IGFBP3	Insulin-like growth factor binding protein 3	0.080		
IGFBP4	Insulin-like growth factor binding protein 4		0.082	0.117
IGFBP5	Insulin-like growth factor binding protein 5		0.055	0.534
IGFBP6	Insulin-like growth factor binding protein 6		0.382	0.640
IGFBP7	Insulin-like growth factor binding protein 7		0.457	0.059
IGFBPL1	Insulin-like growth factor binding protein-like 1		0.549	0.142
GHSR	Growth hormone secretagogue receptor, transcript variant 1a	0.061		
GHSR	Growth hormone secretagogue receptor, transcript variant 1b		0.567	0.751
GHRL	Ghrelin/obestatin preprohormone		0.142	0.205
INS	Insulin		0.279	0.769
INSR	Insulin receptor, transcript variant 1	0.082		
SLC2A4	Solute carrier family 2, member 4	0.020		
SLC2A4RG	SLC2A4 regulator		0.049	0.662
GYS1	Glycogen synthase 1 (muscle)		0.295	0.624
GYS2	Glycogen synthase 2 (liver)		0.176	0.412
PYGL	Phosphorylase, glycogen, liver		0.159	0.573
PYGM	Phosphorylase, glycogen; muscle		0.214	0.570
LIPE	Lipase, hormone-sensitive		0.343	0.618
LPL	Lipoprotein lipase		0.550	0.456
TNF	Tumor necrosis factor (TNF superfamily, member 2)		0.559	0.035
TNFRSF1A	Tumor necrosis factor receptor superfamily, member 1A		0.414	0.065
TNFRSF1B	Tumor necrosis factor receptor superfamily, member 1B		0.045	0.373
IL4	Interleukin 4, transcript variant 1		0.537	0.568
IL4	Interleukin 4, transcript variant 2		0.171	0.067
IL4R	Interleukin 4 receptor, transcript variant 2		0.015	0.585
IL4R	Interleukin 4 receptor, transcript variant 1		0.106	0.048
IL6	Interleukin 6		0.158	0.205
IL6R	Interleukin 6 receptor, transcript variant 1		0.010	0.339
IL10	Interleukin 10	0.058		
IL10RA	Interleukin 10 receptor, alpha		0.474	0.118
IL10RB	Interleukin 10 receptor, beta		0.151	0.511

**Table 4:** Post-hoc analyses indicating fold differences for significant interactions, group, and time effects.

	p-value	Pre	Post-1	Post-6	Post-24
Group					
GH2 var. 3	0.059	1.059	1.055 *	1.012	1.018
GH2 var. 4	0.071	1.064 *	1.082 **	1.042	1.057
SSTR2	0.020	1.374 **	1.588 **	1.256	1.423 **
IGFBP5	0.055	1.066 **	1.055 *	1.005	1.046
SLC2A4RG	0.049	1.153 *	1.213 **	1.075	1.115
TNFRSF1B	0.045	1.221 **	1.361 **	1.210	1.383 **
IL4R	0.015	1.060 **	1.123 **	1.052	1.063
IL6R	0.010	1.185 **	1.167 **	1.165 **	1.120 **
IL10	0.024	1.767 **	2.125 **	1.480	1.577
**Time**					
*CON*					
IGF1	0.024		1.019	1.002	-1.017
IGFBP7	0.059		1.050	-1.125	-1.036
TNF	0.035		-1.18 ♦	1.029	-1.088
TNFRSF1A	0.065		1.006	1.032	-1.175 ♦
IL4	0.067		-1.023	-1.051 ♦	-1.056 ♦
IL4R	0.048		-1.270 ♦	1.058	-1.182
*PD*					
IGF1			-1.017	-1.032	-1.096 ϕϕ
IGFBP7			1.137	-1.085	-1.047
TNF			1.024	1.177	-1.124
TNFRSF1A			1.064	-1.049	-1.147
IL4			1.007	-1.052	-1.024
IL4R			-1.166	-1.025	-1.122

Significance of the overall effect/interaction are presented in the p-value column. Simple comparisons were performed to determine significant group differences at each respective time point. A significant time effect was followed up with comparisons of fold differences at Post-1, Post-6, and Post-24 to Pre.

*Denotes group differences at respective time point (p<0.10)

**Denotes group differences at respective time point (p<0.05)

ϕϕ Denotes difference from Pre for PD (p<0.05)

♦Denotes difference from Pre for CON (p<0.10).

### Bioassays

**Fig 1:**
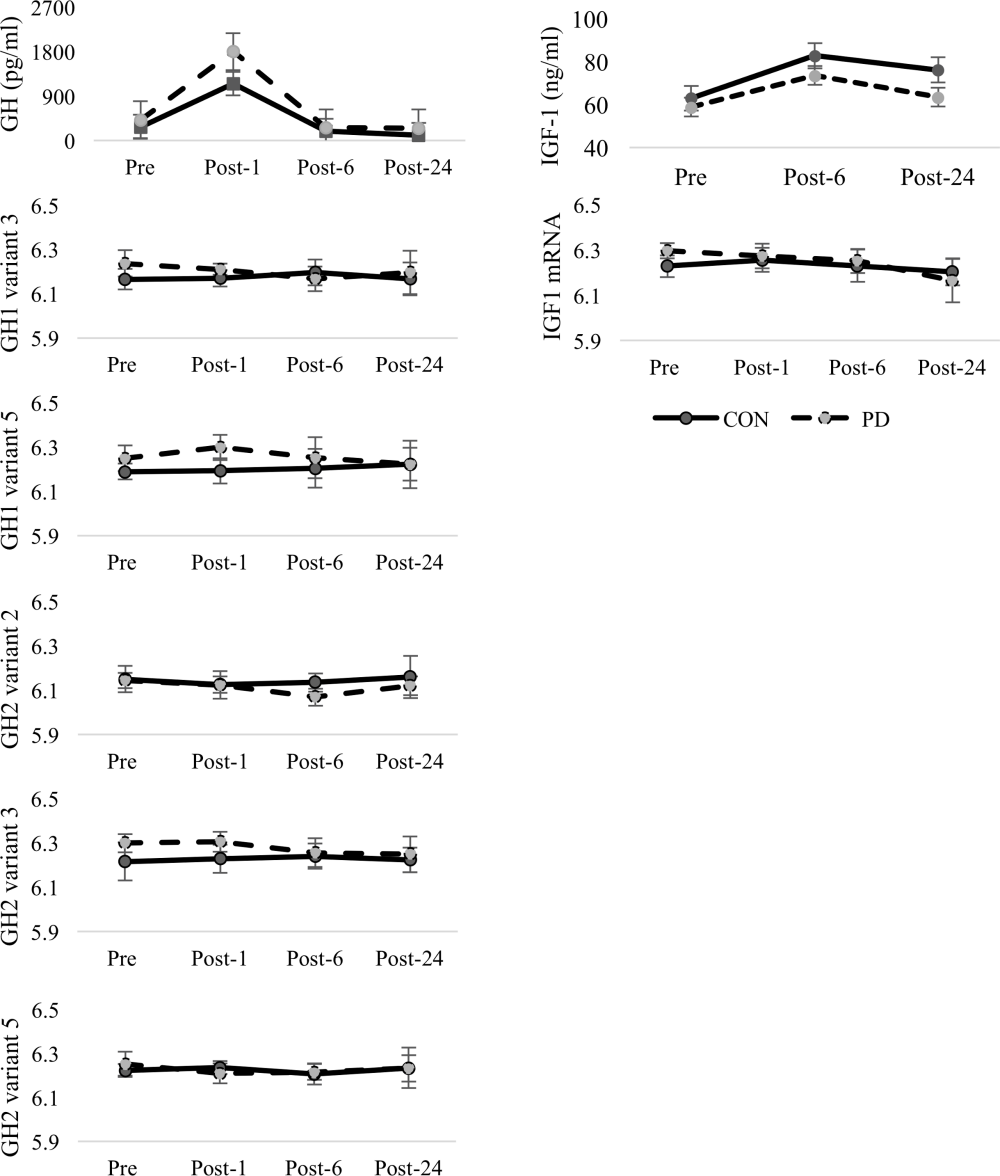
GH and IGF-1 protein concentrations. Comparison of mean (±SD) protein concentrations of GH and IGF-1 as well as the corresponding changes in GH and IGR1 mRNA expression between groups at Pre-, Post-1, Post-6, and Post-24. IGF-1 values at Post-1 were not measured. No significant interactions, group, or time effects were observed for mRNA.

**Fig 2:**
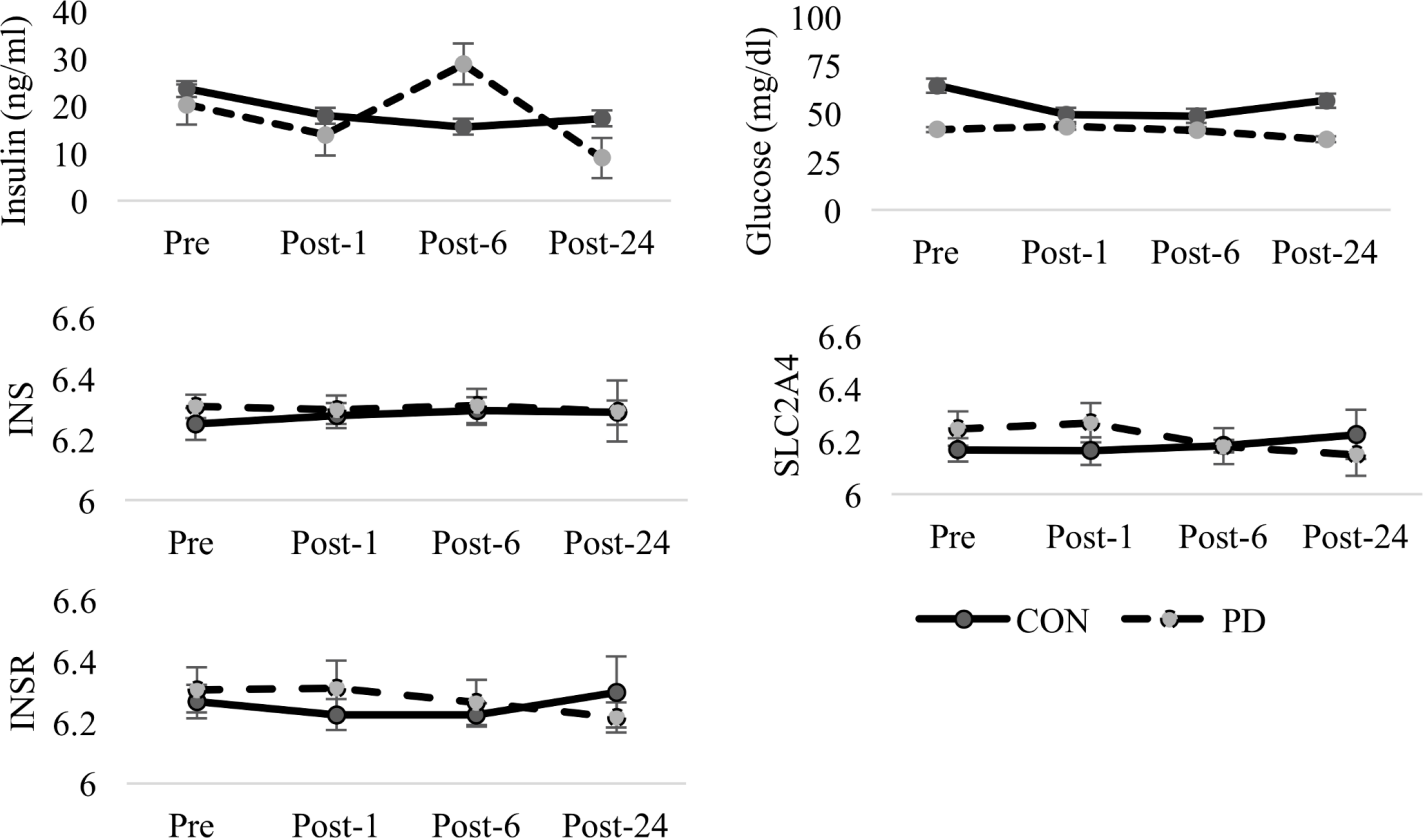
Glucose and insulin protein concentrations with corresponding INS and INSR mRNA expression between groups. Comparison of mean (±SD) protein concentrations of glucose, insulin, INS and INSR mRNA expression between groups at Pre-, Post-1, Post-6, and Post-24. No significant group, or time effects observed for INS. An interaction between group and time for INSR approached significance (p = 0.082) while a significant interaction was observed for SLC2A4 (p = 0.20).

**Fig 3:**
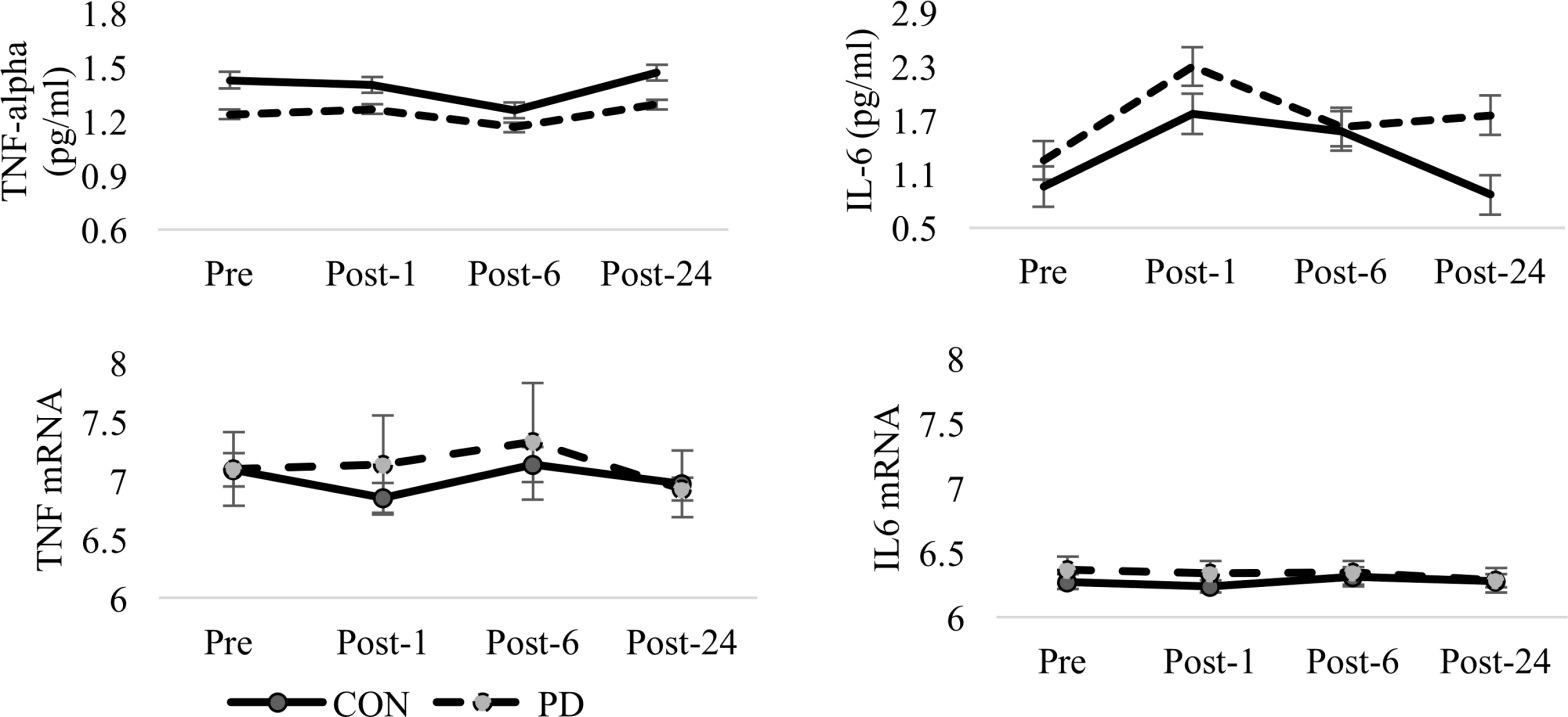
TNF-α, IL-6 and corresponding mRNA between groups. Comparison of mean (±SD) protein concentrations and corresponding values of mRNA expression for TNF-α and IL-6 between groups at Pre-, Post-1, Post-6, and Post-24. No significant group effects were observed for TNF or IL6 mRNA. A main effect for time was observed for TNF mRNA (p = 0.035) but not for IL6 mRNA.

No significant differences were observed in GH AUC between groups (t = 0.473, *df* = 8, p = 0.649). No differences were observed in the ΔGH (t = 0.782, *df* = 8, p = 0.457) or GH_rec_ (t = 0.352, *df* = 8, p = 0.734) between groups, representing a similar exercise-induced GH response for both groups. While body composition measures (including %fat, fat mass, and lean mass) were not statistically significant between the PD and CON, the apparent increase in lean mass within the PD group may contribute to these null findings. No differences were observed for IGF-1 AUC calculations (t = 0.175, *df* = 8, p = 0.865). Graphical representations of the measured GH and IGF-1 concentrations are provided in Fig 1. Graphical representations of glucose and insulin are shown in Fig 2 while TNF-α and IL-6 are presented in Fig 3. There were no significant group differences in IL-6 AUC (t = 0.713, *df* = 8, p = 0.496) or TNF-α AUC (t = 0.713, *df* = 8, p-value = 0.496) in response to the acute moderate intensity exercise protocol. No group differences were observed for ΔIL-6 (t = 0.873, *df* = 8, p = 0.408), TNF-α_rec_ (t = 0.43994, *df* = 8, p = 0.672), or IL-6_rec_ (t = 1.44, *df* = 8, p = 0.1876, ΔTNF-α (t = 0.397, *df* = 8, p = 0.702). Coefficient of variation for GH, IGF-1, TNF-α, and IL-6 bioassays were calculated as 8.0±6.5%, 5.9±5.8%, 3.6±4.3 and 5.2±7.6 respectively.

### Growth hormone and IGF-1 related mRNA

**Fig 4:**
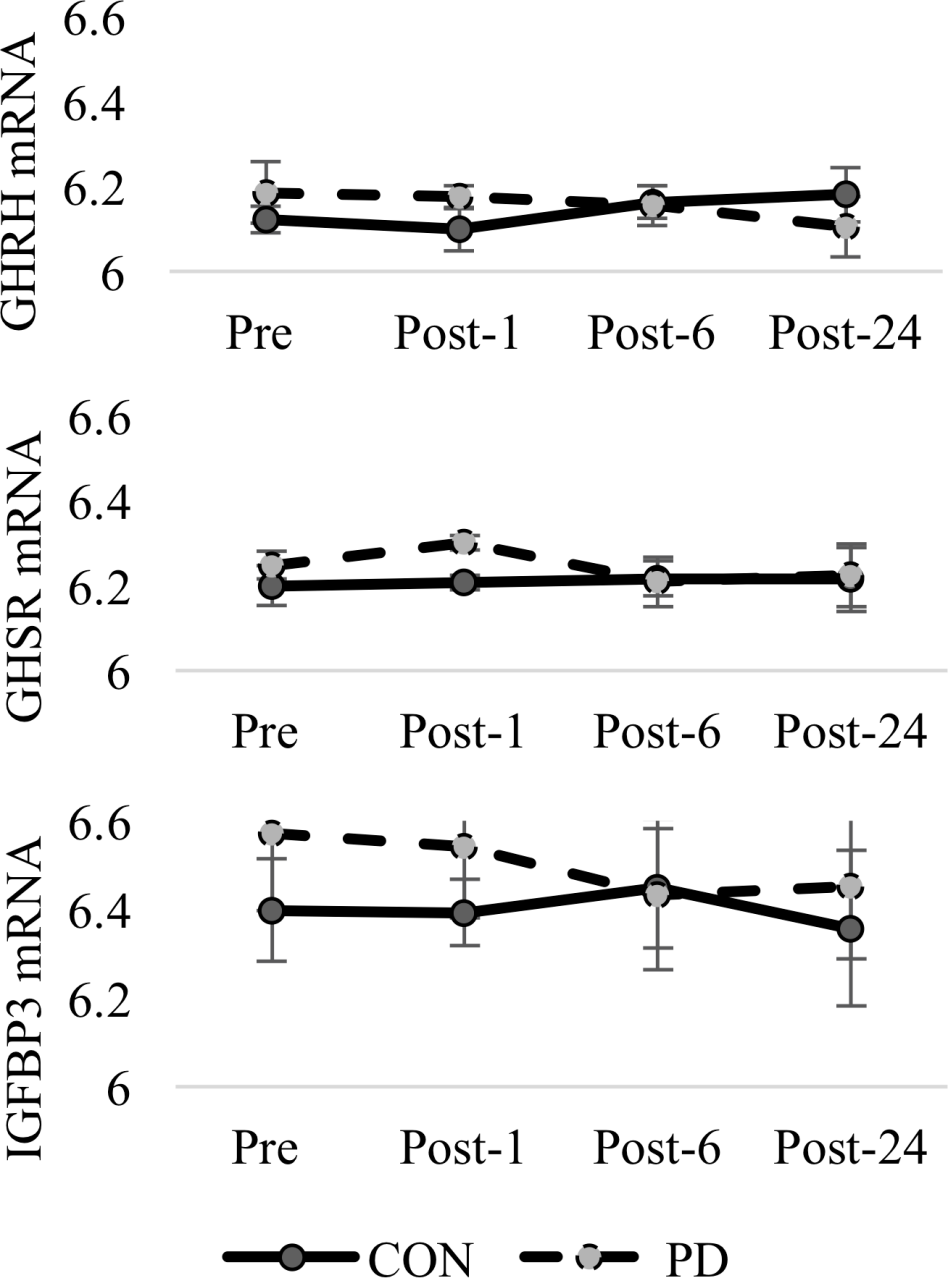
GHRH, GHSR, and IGFBP3 mRNA between groups. Comparison of mean (±SD) mRNA expression for GHRH, GHSR, and IGFBP3 between groups at Pre-, Post-1, Post-6, and Post-24. (Reported values are log-transformations).

No differences were observed for GH1 variant 3 (group comparison p = 0.263; time comparison p = 0.842) or GH1 variant 5 (group comparison p = 0.117; time comparison p = 0.799). No effect for time was observed for GH2 variant 2, GH2 variant 3, or GH2 variant 4, however, group differences for GH2 variant 2 (p = 0.059) and variant 3 (p = 0.070) approached significance. A significant interaction between group and time was observed for GH releasing hormone (GHRH) mRNA (p = 0.008) but no effect for group or time were reported for GHRH receptor (GHRHR) variant 1, or GHRHR variant 2. Somatostatin receptor 2 (SSTR2) was significantly different between groups (p = 0.020), however, no differences were observed for any of the other SSTRs. A near significant interaction between group and time was observed for GH secretagogue receptor (GHSR) transcript variant 1a (p = 0.061) while no differences were observed for GHSR variant 1b. Visual presentation of the interaction between group and time for GHRH and GHSR are provided in Fig 4.

A significant time effect for IGF1 mRNA (p = 0.024) was observed, however, no other differences were observed for IGF1 or IGF1 receptor (IGF1R). The interaction between group and time for IGFBP3 trended toward significance (p = 0.080) and is presented in Fig 4. Similarly, a near significant group difference (p = 0.052) was observed for IGFBP5. Changes in IGFBP7 trended toward significance over time (p = 0.059). No effect for group or time was observed for the ghrelin/obestatin (GHRL) encoding gene.

### Substrate mobilization and transport related mRNA

While a near significant interaction between group and time was observed for insulin receptor (INSR) mRNA (p = 0.082), no significant effects were observed for insulin (INS) expression. Neither liver glycogen synthase (GYS1) nor muscle glycogen synthase (GYS2) mRNA was differentially expressed between groups or across time. Similarly, no differences in liver glycogen phosphorylase (PGYL) mRNA (p = 0.159) or muscle glycogen phosphorylase (PYGM) (p = 0.176) were observed between groups. Neither lipoprotein lipase (LPL) mRNA (p = 0.550) or hormone sensitive lipase (LIPE) mRNA (p = 0.343) were different between groups.

Genetic transcripts associated with glucose transport are part of the solute carrier 2 (SLC2) family. A significant interaction between group and time (p = 0.020) was observed for SLC2A4, which encodes glucose transporter type 4 (GLUT-4) mRNA and a significant group effect (p = 0.049) was observed for the SLC2A4 regulator.

### Inflammatory related mRNA

Tumor necrosis factor (TNF) mRNA (p = 0.035) was altered across time while differences in TNF receptor superfamily (TNFRSF) member 1a (TNFRSF1A) trended toward significance (p = 0.065) across time. TNFRS1B was differentially expressed between groups (p = 0.045) and a near significant main effect for time was observed for both interleukin (IL) 4 (IL4) variant 2 (p = 0.067) and IL-4 receptor (IL4R) variant 1 (p = 0.050). These findings were coupled with differential expression of IL4R variant 2 between groups (p = 0.015). While no differences in IL-6 (IL6) mRNA were observed between groups or across time, a significant group difference was observed for IL6 receptor (IL6R) variant 1 (p = 0.010); indicating elevated expression of IL6R mRNA in the PD group compared to CON. This difference was coupled with a significant increase of IL10 in PD compared to CON (p = 0.024). No differences in IL10 receptor (IL10R) alpha (IL10RA) or beta (IL10RB) were reported between groups or across time.

## Discussion

With knowledge of physiological differences in GH and IGF-1 secretion in healthy controls compared to individuals with T2D, the purpose of this study was to examine differences in exercise-induced gene expression specifically related to the GH and IGF-1 pathways of healthy and PD AA individuals following an acute bout of moderate intensity exercise. Analysis of GH AUC and IGF-1 AUC suggests that overall release of both hormones was similar between the two groups in response to the exercise stimulus. Trends in the mean concentrations of GH following exercise suggest that the exercise-induced GH response may be higher in the PD group compared to CON. Nevertheless, this observation was not supported by a statistical comparison of the exercise-induced GH response immediately post-exercise (ΔGH). The PD group performed slightly more work (Kcal expended), had slightly higher lean body mass and slightly lower percent body fat compared to the CON group, all of which could potentially contribute to slightly higher GH output [32]. This study was designed specifically to investigate changes in gene expression and the blood sampling paradigm was chosen to maximize the chances of capturing these changes. We recognize that the lack of a GH profile and pulsatility information limits the conclusions that can be gleaned from the current exercise-induced GH values and the comparison of this data to many of the previously published results on exercise-induced GH responsiveness. Because the timing of the blood draws likely did not catch the exercise-induced GH increase, we are unable to accurately compare how potential differences in peak GH output, or the 24-hr GH AUC, may have altered gene expression for GH or inflammatory related transcripts between PD and CON. However, potentially significant differences in either of these values would alter the subsequent regulation of gene expression.

While no significant group differences in GH output were observed in the 24-hours following exercise, GH2 mRNA was differentially expressed between the two groups. These differences appear to be present prior-to (Pre) and immediately following exercise (Post-1) with the PD group having approximately 5% higher expressions of GH2 mRNA compared to CON. This data suggests that both elevated GH mRNA and GH release may occur in PD compared to CON in response to an acute exercise stimulus, although these findings should be interpreted with caution given the small number of subjects in the current study. As previously mentioned, the ability of GH to affect insulin sensitivity has been well established [17]. Both well- and poorly- maintained T2D have shown an altered exercise-induced GH response compared to healthy controls [33–35]. More specifically, diabetics with well-controlled blood glucose (blood glucose values more like healthy individuals), elicited a blunted GH response compared to individuals with poorly-controlled diabetes [34, 35]. Our findings suggest that the exercise-induced GH response in PD individuals would more likely correspond to the GH response observed in individuals with well-controlled diabetes and this is likely due to the fact that most of these individuals had fasting blood glucose values that were only slightly elevated, suggesting they were very early in the progression toward T2D. In addition, these observations further support the idea that an altered exercise-related GH response in PD precedes altered resting serum GH concentrations typically seen in T2D [36].

A significant interaction between group and time for GHRH gene expression was coupled with GH protein concentrations that appeared to be similar between the two groups outside of the immediate response following exercise. An elevated expression of GHRH in PD during Pre and Post-1 was paired with elevated expression in CON at Post-24. This suggests that an exercise stimulus differentially effects expression of GHRH mRNA and this difference could be due to various factors. Since GHRH is the primary pulse generator for GH release, PD individuals may actually have consistently increased output of GH compared to CON or differential pulsatility profiles for GH. However, because we do not have sustained 24 hour profiles of GH either in response to the exercise stimulus or in an unstimulated 24-hour period for comparison, it is virtually impossible to tell if elevated GHRH mRNA results in significantly more GH output in PD. Potentially higher GH outputs in the PD group suggests this may be the case, but further investigation is necessary to confirm this finding. It is also possible that PD produce more GHRH but that these individuals express alterations in hypothalamic regulatory mechanisms that inhibit its release or that differences in somatostatin concentrations or sensitivity exist. Of course, as is the case with all assessments of mRNA, increased levels of mRNA do not automatically result in greater protein expression and it is possible that elevated destruction of GHRH mRNA or lower rates of translation, necessitate higher levels of GHRH mRNA in PD to maintain output of GH at the levels needed for proper physiological function. The increased expression in CON at Post-24 may also represent a preparatory response for future exercise events and the lack of this response in PD suggests that these individuals are not responding as expected and mounting the necessary production for future exercise.

Although SST mRNA expression was not different between groups, elevation of SSTR2 mRNA expression in the PD group at Post-1 and Post-24 suggests a counter regulatory feedback mechanism may be present. While evidence from this study is inconclusive and measurement of GHRH, somatostatin and somatostatin receptor proteins would be needed before we could support this theory, an upregulation in SSTR2 in response to an acute bout of moderate intensity exercise in PD may indicate a shift in sensitivity as a counter regulatory mechanism to an altered exercise-induced GH response. Ultraradian oscillations in both hypothalamic GHRH and somatostatin are known to regulate GH secretion [37] and higher concentrations of somatostatin reduce the responsiveness of pituitary somatotrophs to GHRH. This sensitizes these somatotrophs to future GHRH release and suggests that while an upregulation of SSTR may precede an increase in somatostatin receptor protein concentration, the counter regulatory mechanisms associated with an altered exercise-induced GH response in PD may paradoxically increase the sensitivity of the anterior pituitary to future GHRH release [38].

While the findings of IGFBPs are interesting, it is important to consider not only the physiological role related to the extension of the half-life of IGFs [39], but the other roles in biological function independent of being bound to IGF-1 [39]. IGFBP-3 is the most abundant IGFBP in circulation and high IGFBP-3 may be a risk factor for insulin resistance in the development of T2D [39] irrespective of IGF-1 levels [40]. In addition, low IGFBP-1 and IGFBP-2 coupled with elevated IGF-1, may represent compensatory mechanisms in response to increasing insulin resistance [39]. We observed non-significant group differences in IGF1 and IGF receptor-1 (IGF1R) mRNA that were coupled with significantly elevated levels of IGF binding protein-5 (IGFBP5) mRNA in the PD group. We also observed a significant interaction between group and time for IGFBP3 mRNA which may correspond to the previously reported alterations in IGFBP-3 concentrations in T2D [39]. Wheatcroft and Kearney [41] reviewed the role of IGF-dependent and IGF-independent actions of IGFBP-1 protein and IGFBP-2 protein on regulating metabolic homeostasis. Specifically, they discuss the inverse relationship between IGFBP-1 concentrations and insulin. They also discuss how IGFBP-1 protein concentrations and insulin sensitivity might serve an inhibitory effect of hyperinsulinemia on hepatic IGFBP-1 synthesis from the liver; suggesting IGFBP-1 transcription in insulin resistant individuals is sensitive to plasma insulin levels. We failed to observe any difference in INS mRNA between groups but the insulin response following exercise (Post-6) in the PD group may shed light on an interesting relationship between the progression of diabetes and differing glucoregulatory responses between PD and CON following exercise.

O’Gorman et al. [42] previously observed increases in GLUT4 protein content in T2D individuals despite that SLC2A4 expression was unchanged following acute or chronic exercise in either T2D or obese non-diabetic individuals. Exercise training increased insulin-mediated glucose disposal in the individuals with T2D irrespective of the alterations in the insulin-signaling cascade and GLUT4 protein concentration [42]. The most notable difference between the samples of O’Gorman et al. [42] and our study were the differences in BMI; the PD group from our sample matched closely to the non-diabetic obese individuals of O’Gorman et al., while our CON group had a notably lower BMI and the T2D group from O’Gorman et al. was significantly higher (BMI: 35.8). The difference in our observations from those previously reported may be due to sample size or may suggest that other factors, such as weight and body fat percentage, may contribute to SLC2A4 expression. While the samples are not directly comparable and GLUT-4 protein content was not measured in the current study, we did observe differentiated expression of SLC2A4. Elevated expression of SLC2A4 at Pre and Post-1 time points was eliminated at Post-6 and Post-24. Previous evidence indicates that elevated insulin activity increases transcription of SLC2A4 [43]. While we did observe an increase in insulin at Post-6 in PD, SLC2A4 gene expression remained similar between PD and CON at both Post-6 and Post-24. Exercise training is known to increase skeletal muscle GLUT-4 concentrations [44, 45], which quickly translates to skeletal muscle GLUT-4 protein concentrations [46, 47]. It is difficult to tease apart all the potential influences on SLC2A4 gene expression since individuals were allowed to eat after the exercise session (prior to the Post-6 draw) and food consumption was not monitored for the remainder of the day.

While the changes in TNF-α and IL-6 protein were not statistically different between groups, it does appear as though elevated concentrations of IL-6 were present in the PD group compared to CON. However, these observations were not corroborated by differences in either TNF or IL6 mRNA. While the exercise-induced changes in TNF and TNFRSF1A mRNA represent an increased expression of the inflammatory genes that therein correspond to the inflammatory response that may be expected in aerobically untrained individuals following an exercise stimulus, no changes in IL6 mRNA were observed within the 24 hours. Interestingly, the increased expression of TNFRSF1B in PD further suggests that although TNF-α protein concentration may be lower in the PD group, they may also have a more sensitive inflammatory response to an exercise stimulus. This is further corroborated by elevated expression of IL6R mRNA and lower expression of IL4 mRNA in PD compared to CON throughout the 24 hours. Elevations in IL4R were coupled with increased expression of IL10 mRNA. Nevertheless, we did not assess protein levels for all these inflammatory markers and thus, we can’t address the balance of the pro- versus anti-inflammatory profile that resulted in each group in response to the exercise stimulus. However, it is crucial to point out that the findings of increased pro-inflammatory and reduced anti-inflammatory gene expression followed the pattern one would expect to see in PD compared to CON given published findings related to inflammation and diabetes [48].

This study has several limitations. As pointed out earlier, a full GH profile would permit additional descriptive indices to be compared and for measures of variability and complexity to be assessed. To adequately address any exercise-induced changes in GH secretion, blood samples should be taken at least every 10–15 minutes for 2 hours or more. While the current analyses indicate that the overall GH response was not different between groups, these findings are limited by low sample size and poor statistical power that is inherent in a pilot study of this nature. In addition, these limitations prohibited us from appropriately comparing concentrations of TNF-α and IL-6 between groups and across time. We asked that individuals eat a standardized breakfast approximately 90–120 minutes prior to arriving at the lab for the exercise session and that they eat this exact same standardized breakfast prior to arriving at the lab for the 24 hours post exercise (Post 24) blood draw. We also asked that individuals be at least 2 hours post-prandial for the 6 hour post-exercise blood draw (Post-6). However, we did not monitor food intake or ask individuals to report foods that were eaten during the 24-hour period other than the standardized breakfast. Thus, it is possible that some of the gene expression changes observed in the current study were related to either acute dietary intake differences between the groups, or potentially reflected chronic differences in dietary habits between the 2 groups. While selectively including African American’s into the study was a methodological decision related to diabetes risk, it limits the generalizability of the findings to other ethnic groups. Although we expect that similar patterns would exist across all ethnicities, genetic differences that increase susceptibility for various diseases in certain ethnic groups may further compound the signaling differences between PD and healthy controls in response to exercise. In addition, we recognize that the differences between the 2 groups may be limited by several other factors; 1) individuals in the pre-diabetes group were screened in to this group based on one fasting blood glucose value only, 2) none of these individuals had ever been told they were pre-diabetic by a physician suggesting that the PD individuals in our study were in the beginning of the disease process and 3) family history for T2D was similar between the 2 groups.

In summary, we observed elevated levels of GH2 mRNA in the PD before and immediately following exercise that do not directly correspond to the observed changes in GH protein concentrations. While our findings, from a statistical perspective, indicate that the GH and IGF-1 protein response to an acute bout of exercise did not differ between groups, additional studies are warranted. The seemingly incongruent findings between GH and IGF-1 related mRNA expression and the GH and IGF-1 protein concentrations observed in the blood, pose interesting questions regarding the regulatory mechanisms associated with these transcripts in response to an acute exercise bout. While the regulation of associated proteins may not represent physiologic strain, the differences in associated mRNA do suggest that some stressor, possibly the pre-diabetic state, moderates resting and exercise-related effects on GH-axis gene expression. Given the known benefits of exercise for treating and managing T2D, these findings further highlight the important role that exercise plays in the treatment of this disease. Furthermore, these findings provide context to how a 60-minute moderate-intensity exercise affects disease-related physiological mechanisms at the lowest levels of physiological organization. Additional research is warranted and we recommend that specific research questions target how different exercise intensities and durations affect gene expression in T2D. For instance, it may be that different types of exercises are more beneficial to mitigating the effects of disease in this population. Such findings would provide evidence to support changing/adapting current guidelines to more dynamically target the treatment of disease through exercise.

## Supporting information

S1 File Medical screening.doc. (DOC)

## Abbreviations

AA: African American
FBG: Fasting blood glucose
HbA1C: Glycated hemoglobin
OGTT: Oral glucose tolerance test
PBMC: Peripheral blood mononuclear cell
PD: Prediabetic
RT qPCR: Real-time quantitative polymerase chain reaction
T2D: Type 2 Diabetes
