# Supplementary material for: The effects of an acute exercise bout on GH and IGF-1 in prediabetic and healthy African Americans: A pilot study investigating gene expression
Source: PLoS One. 2018 Jan 19;13(1):e0191331. doi: 10.1371/journal.pone.0191331 (PMC5774763; doi:10.1371/journal.pone.0191331)
Supplement: S1 File — (DOC) [file pone.0191331.s001.doc]

**MEDICAL QUESTIONNAIRE**

The purpose of this questionnaire is to determine if you have any physical limitations that may exclude you from participation in this investigation. All information will be kept completely confidential.

**A. Personal Information**

Date of Birth: ___________ Gender: ________

Address/Street: ________________________________________________

City: _______________ State: ________ Zip Code: ________

Phone (H): _________________ Phone (w): ________________

Phone (cell): ________________ E-mail: ___________________

Best way to reach you and when?_____________________________________

1. When was your last physical exam? ___________________

2. Please list any serious or chronic illnesses of which you are aware.______________________

3. Please list any allergies to medications, foods, or other substances. __________________________________________

4. Please list any medication you have been on or presently take

Type Dosage/Frequency How Long? Why?

**B. Medical History**

1. Illnesses---Please check if you have had any of the following:

Illness Present Past Dates

Heart attack

Anemia

Asthma

Epilepsy

Lung disease

Stroke

Gout

Diabetes

Hypoglycemia

Rheumatic fever

Heart murmur

Hernia

2. Symptoms---During the last 12 months, have you experienced:

Condition Yes No

High blood pressure

Swelling of hands and feet

Pain or cramps in legs

Orthopedic problems

Musculoskeletal problems

ECG abnormalities

Blurred vision

Chest pain/pressure

Shortness of breath

Unusual fatigue

Dizziness/light headed

Significant weight change

High cholesterol

Numbness in limbs or face

3. Is there any chance you are pregnant?

4. Hospitalizations---List the dates and the reasons for hospitalizations for any significant illness.

Date Diagnosis

1.

2.

3.

**C. Family History**

1. Is your father living? Yes No If not, age at death and cause.

2. Is your mother living? Yes No If not, age at death and cause.

3. Has you father, mother, grandparents, or siblings had:

***Condition Yes No Who?***

High blood pressure

Stroke

Heart attack (<50 yrs)

Heart attack (>50 yrs)

Diabetes

Cardiovascular disease

Other

**D. Social History**

1. Have you ever smoked? **Yes No**

If yes, when did you quit? ________

If yes, how long did you smoke? ________________ (in yrs or months)

If yes, approximately how many cigarettes did you smoke per day? _______

2. Do you drink alcohol? **Yes No**

If yes, think about the past month, how many alcoholic beverages have you consumed in each of the categories below,

Beer _____ per day _____ per week _____ per month

Wine _____ per day _____ per week _____ per month

Liquor _____ per day _____ per week _____ per month

**E. Menstrual & Hormonal History (Women Only)**

1. How old were you when you started your menstrual periods (yrs of age)? _________

2. When was the first day of your las period (month/day)? _________

3. On average, how many days are there between your menstrual periods _________

(ie. 21, 28, 32 days, etc)

4. How many menstrual periods have you had in the last 12 months? _________

5. Does your cycle vary more than 1-2 days per month **Yes No**

6. When do you expect your next menstrual period to start (month/day)? _________

7. Are you currently taking hormones (eg. Birth control pills, estrogen

therapy, etc) for any reason? **Yes No**

If yes; type of hormone ________________________

If yes; How long have you been taking this hormone?

________________________

8. Is there any chance you are currently pregnant? **Yes No**

9. Do you have plans to become pregnant in the near future? **Yes No**
